# A qualitative study of father involvement with their young children in mainland China

**DOI:** 10.3389/fpsyg.2025.1542136

**Published:** 2025-04-24

**Authors:** Yang Liu, Mingchun Guo, Cassandra K. Dittman, Ying Zheng, Divna M. Haslam

**Affiliations:** ^1^School of Psychology, Fujian Normal University, Fuzhou, China; ^2^Parenting and Family Support Centre, School of Psychology, The University of Queensland, Brisbane, QLD, Australia; ^3^School of Psychology, Central China Normal University, Wuhan, China; ^4^School of Health, Medical and Applied Sciences, Central Queensland University, Bundaberg, QLD, Australia; ^5^Psychological Counseling Center, Liming Vocational University, Quanzhou, China; ^6^School of Public Health, The University of Queensland, Brisbane, QLD, Australia

**Keywords:** father involvement, Chinese fathers, semi-structured interviews, reflexive thematic analysis, grandparents

## Abstract

**Introduction:**

The voices of Chinese fathers regarding their involvement with their young children remain largely absent in the existing research. Thus, it is critical to explore subjective experiences of and possible influences on Chinese fathers’ involvement with children from their point of view.

**Methods:**

This study conducted semi-structured interviews with 35 Chinese fathers of preschoolers. Reflexive thematic analysis was used to analyze the interviews.

**Results:**

Chinese fathers both adhered to traditional paternal roles and increased proximity to their children while serving as maternal coparenting partners. Father involvement was related to intrapersonal, familial, and contextual factors. Intrapersonal factors included taking responsibility for being a father, optimizing children’s development and future, beliefs about parental roles, and fulfillment and joy versus tiredness and boredom. Familial factors include maternal availability for childcare and advocacy, the child’s requests and rejections, and the grandparent’s involvement and impediments to father involvement. Contextual factors include fathers’ occupational demands, networking demands, and invitations as well as opportunities for father involvement from school and community.

**Discussion:**

The findings supported that multi-layered factors jointly influence how fathers are involved with their children. Yet, different from Western models of fathering focusing solely on child and mother characteristics, the role of grandparents was highlighted in Chinese fathers’ narratives, reflecting cultural influences on family dynamics. This highlights the need to consider the potential impact of grandparents in China and many other Asian countries, where multigenerational living is common. Additionally, the identified factors can guide the design of family programs and family-friendly policies to facilitate father involvement.

## 1 Introduction

The last few decades have seen a growing interest in fathers and fatherhood from social scientists (Lamb, 2000). There is considerable evidence that father involvement is associated with positive outcomes for children (Cano et al., 2019; Sarkadi et al., 2008; Torres et al., 2014; Zhang et al., 2019). As fathers increasingly spend more time with their children (Craig et al., 2010; Pleck and Pleck, 1997; Pleck and Masciadrelli, 2004), expectations for paternal roles have shifted from the more traditional tasks of moral teachers, breadwinners, and male role models, to a more modern role as nurturing co-parents actively involved in all aspects of childcare and development (Lamb, 2000). However, most existing research has focused on fathers in the Global North societies with few studies carried out in the Global South contexts (Li et al., 2021). Although Chinese fathers are one of the largest ethnic groups of fathers globally, there is a relative paucity of research focusing on Chinese fathers. In addition to the sheer number, Chinese fathers, under the influence of a collectivistic culture as opposed to an individualistic culture, might have unique experiences and challenges when involved with their children and families. Therefore, more research attention should be directed toward Chinese fathers and their role in parenting to determine if findings from societies within the Global North apply to a Chinese context. The present study used a qualitative research approach to exploring Chinese fathers’ perceptions of involvement with their preschool-aged children and the factors that facilitate or hinder their involvement.

### 1.1 The role of fatherhood in China

Parenting beliefs and behaviors are profoundly influenced by traditional culture as well as social contexts and changes; thus, taking a historical perspective to understand Chinese fatherhood is essential (Abbott et al., 1992). The traditional Chinese cultures, including Confucian, Taoist, and Buddhist thoughts, as well as cultures from ethnic minorities, influence Chinese families and their parenting values (Li and Lamb, 2013). Confucian ideology legitimated the patriarchal culture in the traditional Chinese family (Ebrey, 2003). Under the patriarchal traditions, gender roles were differentiated in the traditional Chinese family (Shek, 2006). Men were regarded as “the master of the family” (*yi jia zhi zhu*; Shek, 2006) and in charge of “external affairs,” providing, and decision making (Li and Lamb, 2015). As the popular Chinese proverb goes, “Men are chiefly responsible for activity in society, while women are responsible for the home” (*nan zhu wai, nv zhu nei*).

For traditional Chinese parents, *jiaoyang* is their prime parental responsibility (Chen et al., 2012). This translates as educating, cultivating, and teaching (*jiao*) and rearing, raising, and feeding (*yang*; Cao and Lin, 2019; Chen et al., 2012). In line with gender role differentiation, paternal and maternal roles in *jiaoyang* are also differentiated (Li and Lamb, 2013). The responsibility of *yang* for fathers is financial support and material provision for the family, but childcare and nurturing, which are also part of *yang*, are left to mothers (Cao and Lin, 2019). Due to fathers’ presumed superiority in morality and education in tradition, they typically take more responsibility for educating and disciplining (*jiao*) their children, though mothers are also involved in managing children’s misbehavior, especially when children are young (Li and Lamb, 2013). After children reach the “age of reason” (*dong shi*, around the time children begin formal schooling), fathers start to assume roles in imparting social etiquette and moral codes, disciplining children, and setting an example for their children (Li, 2018). The words, such as “The father guides the son” (*fu wei zi gang*) and “To feed without teaching is the father’s fault” (*yang bu jiao, fu zhi guo*), emphasize fathers’ critical roles in jiao (educating).

Since the founding of the People’s Republic of China in 1949, there has been a series of drastic changes in society, policies, and economy, which influenced traditional Chinese family values and culture (Xu, 2016). As the socialist political and economic systems were established, the patriarchal family system was severely challenged (Shek, 2006). For example, the egalitarian policies promulgated in 1954 granted women equal legal rights and encouraged them to pursue opportunities in education and careers (Li and Lamb, 2013). According to the World Bank, Chinese females’ labor participation was 61% in 2023, which is higher than the numbers of many Asian and Western countries, such as Japan, Korea, the US, and Germany (International Labour Organization, 2024). With the implementation of the “reform and opening-up” policy in 1978, China has gradually opened up its international borders. Western values and ideologies, such as individualism and democracy, have been brought into Chinese society and influenced Chinese family values (Ji et al., 2011). In the market economy, the growing trend of mobility driven by employment and the increasing demand for independence among younger generations have also challenged the traditional practices of patrilocal residence and multigenerational living (Li and Lamb, 2015). The proportion of nuclear families also increased (Li and Lamb, 2013). On the other hand, marketization potentially led to the state welfare decrease for the family (e.g., food, accommodation, daycare) and the decline of the state ideology that once focused on promoting gender equality. Economic advancement in recent years has potentially increased cultural self-confidence in China. The traditional culture, such as Confucian thought, has regained people’s attention (Li and Lamb, 2013), which might further influence the Chinese family system and values.

This socio-cultural transformation, high female labor participation, and growth of nuclear families have contributed to some shifts in gender expectations and parental roles in Chinese society. Contemporary Chinese fathers are now more involved with their children than their predecessors (Li, 2020a; Li and Lamb, 2013). They also play more roles in parenting, including being a provider, an educator, a caregiver, a helper, a communicator, a playmate, and so on (Lau, 2016; Li and Jankowiak, 2016; Liong, 2017; Xu, 2016). However, the small amount of research conducted indicated Chinese fathers may still prioritize traditional roles when involved with children, compared to their Western counterparts. For example, Western fathers are frequently noted for their role as playmates (Cabrera and Roggman, 2017; Robinson et al., 2021), possibly due to the cultural emphasis on physical strength, independence, and assertiveness within Western societies (Li et al., 2021). In contrast, being an educator is most frequently mentioned when Chinese fathers describe their parenting roles (Chuang et al., 2013). This may stem from the impact of traditional Chinese culture, which highlights the prime responsibility of fathers in *jiao* (educating) (Chen et al., 2012). Such differences underscore the necessity for extreme caution when applying theories and findings originally developed within Western contexts to Chinese fathers. The interplay of traditional Chinese culture and contemporary social trends has a collective impact on how Chinese fathers are involved with their children.

### 1.2 Factors influencing father involvement from an ecological perspective

As Cabrera et al. (2000, p. 132) suggested, “men do not father in a social vacuum,” many factors potentially influence how fathers are involved with their children. From an ecological perspective, multi-layered and interrelated systems, namely intrapersonal, familial, social, cultural, and other contextual factors, might have effects on fathers’ behaviors and commitment to fatherhood (Doherty et al., 1998; Shek, 2006). For example, Doherty et al. (1998) developed a conceptual model suggesting that fathering can be influenced by (1) his role identification, commitment, skills, experience, and psychological well-being; (2) the mother’s beliefs of and support for father involvement; (3) the child’s characteristics, such as gender, developmental status, and temperament; (4) his relationship with the mother; and (5) contextual factors, including employment, economic factors, cultural expectations, and social support. Support for this model has been found in Western contexts (e.g., Freeman et al., 2008; McLaughlin and Muldoon, 2014; Schoppe-Sullivan and Fagan, 2020).

A few studies have examined the influence of intrapersonal, familial, and contextual factors on father involvement in the Chinese context. With respect to intrapersonal factors, fathers who strongly believe in the important roles fathers play in child development and have higher fathering self-efficacy tend to have higher levels of involvement with their children (Kwok and Li, 2015). At the familial level, for example, both maternal encouragement and discouragement have been found to be related to father involvement with young children (Wang et al., 2019) and adolescents (Zou et al., 2019). At the broader contextual level, work commitments have been recognized as one of the major barriers to father involvement in parenting. The longer fathers worked, the less involved they were in interactions with their children (Wu et al., 2014).

Although these studies have extended our knowledge of factors associated with father involvement in the Chinese context, a systematic understanding of Chinese fatherhood is still lacking. For example, it is unknown to what extent Doherty et al. (1998) conceptual model developed in Western socio-cultural and ecological contexts applies in the Chinese context and whether there are unique factors that may play a role in facilitating or hindering Chinese father involvement due to cultural specificity. Thus, an ecological perspective was adopted in the present study to analyze Chinese fathers’ views about factors related to their involvement in parenting and childrearing.

### 1.3 The present study

Given the noticeable absence of Chinese fathers’ voices in current research, it is critical to explore subjective experiences of and possible factors related to Chinese fathers’ involvement with children from their point of view. Thus, two research questions were examined in the present study: (1) How are Chinese fathers involved in parenting? (2) Which factors relate to their involvement? These research questions were explored qualitatively using semi-structured interviews, which are instrumental in yielding detailed insights and enhancing our understanding of Chinese father involvement.

This study conducted interviews with Chinese fathers with at least one child in preschool. The preschool years are a period of rapid development for children, during which parental involvement plays a crucial role. Historically, Chinese fathers have been less involved in childrearing until their children reach the “age of reason” (approximately age six), though the influence of child age on fathering appears to be diminished in contemporary China (Li and Lamb, 2015). The interviews with Chinese fathers of preschoolers offered a unique opportunity to investigate how they are involved in parenting and which factors facilitate or hinder their involvement with their young children. In addition, when children start formal schooling at age six, they typically spend most of their time at elementary school and on homework, leaving less time for family interaction. Focusing on fathers of preschoolers might reduce the impact of formal schooling and homework on father-child interactions.

## 2 Methods

### 2.1 Study design

This qualitative study is part of a mixed-method research project, which aims to explore the relationship between parental beliefs and behaviors and child adjustment in mainland China. The research project included a survey (Liu et al., 2022) and a follow-up semi-structured interview conducted in Fuzhou City (the capital of Fujian province and located on the southeast coast of Mainland China). Inclusion criteria for the survey were mother–father dyads who had at least one child in preschool, aged 2–7 years old and lived in China. The interview sample was a subset of fathers who completed the survey and agreed to participate in an interview. The qualitative study was conducted according to the consolidated criteria for reporting qualitative research (COREQ; see Supplementary material, Tong et al., 2007).

### 2.2 Ethical considerations

Ethical approval was obtained from the first author’s departmental ethics committee (clearance number: 17-PSYCH-PHD-66-AH). Before each interview, participants were advised that they were permitted to withdraw at any stage without penalty or to decline to answer any specific questions. They were assured that all data would be anonymized and stored securely, with access restricted to the main researchers. All participants signed the consent form before the interview. As a token of appreciation, each participant received a ¥50 gift card (approximately USD 7).

### 2.3 Participants

A total of 35 fathers with a preschool-aged child participated in the interview. One father was divorced and did not live with his child, while all others were married. Thus, the divorced father was excluded from the data analysis, and the final sample size was 34.

Fathers ranged in age from 26 to 49 years (*M* = 34.81; *SD* = 4.99). Mothers ranged in age from 24 to 42 years (*M* = 32.79; *SD* = 3.98). Children had a mean age of 4.81 (*SD* = 0.76). Roughly equal numbers of girls and boys (47.1% male) and no non-binary children were represented in the research. The average family size was 4.54 (*SD* = 1.22). Most fathers (88.2%) were employed, and their average weekly working hours were 39.85 (*SD* = 24.47). Twenty-six mothers (74.3%) were employed, and their average weekly working hours were 30.71 (*SD* = 22.38). More than half of fathers (55.9%) and mothers (54.3%) had a Bachelor’s or higher degree. Most fathers (88.2%) reported they could meet their essential expenses, and 35.3% of those had enough money to purchase most of the things they really wanted after paying for essential expenses indicating a financially secure sample. Twenty-five families (73.5%) had at least one grandparent living in the same household.

### 2.4 Procedure

The participants of this interview were recruited from fathers who completed the survey. The survey was introduced to parents of children in four public and seven private preschools. If parents showed an interest, they would receive the survey package, which contained an advertisement for this interview. If fathers were interested in the interview, they could sign up by returning their contact information with the questionnaires. In total, 208 fathers expressed an interest in participating in the qualitative study. The first author randomly selected fathers using a web-based random number generator and contacted them via telephone or message to introduce and set up an interview if the father consented. The format (face-to-face or remote interviews), time, and venue for the interview were based on the participant’s preference. All participants provided informed consent before being interviewed. All interviews were conducted by the first author in Chinese (Mandarin) and audio-taped. Interviews lasted between 13 to 53 min.

The semi-structured interviews followed a purpose-developed interview guide (see Appendix Table 1). The interview guide was developed through a systematic process that included a comprehensive review of existing literature, initial drafting of questions, team discussion and refinements, and pilot testing. Before the formal interviews, the interview questions were pilot-tested with 8 fathers to assess clarity, relevance, and effectiveness. Feedback from the pilot interviews was used to further refine questions. The data collected from the pilot interviews were excluded from the final analysis. The interview guide consists of 10 primary questions and follow-up questions and prompts to elicit more information about fathers’ beliefs and experiences. The questions were related to fathers’ beliefs about their roles, such as their definition of “good father” and the importance of the father role, how they were involved in parenting, and which factors influence their level of involvement. Based on interviewees’ responses to the primary questions, subsequent questions and probes were used for further clarification and elaboration.

### 2.5 Research team and reflexivity

The research team was composed of the first author (YL), who primarily conducted all aspects of this study, with the support of her supervisors (DH, CD, and MG) and a research assistant (YZ). YL, MG, and YZ are Chinese, and DH and CD are Australians who do not speak Mandarin. MG is a father, and DH and CD are mothers. YL and YZ are females who did not have a child. The authors had a diverse mix of backgrounds. YL was raised within a traditional Chinese household characterized by gender-differentiated parental roles: her mother shouldered childcare and nurturing responsibilities, while her father focused on financial provision and was less engaged in daily parenting practices. This experience prompted YL to focus on the diverse involvement patterns of contemporary Chinese fathers and associated factors, comparing them with the roles of traditional Chinese fathers during data collection and analysis. YL acknowledged the possible influence of personal experience on the research and engaged in regular reflexive practices. Furthermore, the integration of the literature review and the collaboration of the research team, offering multiple individual, cultural, and professional perspectives, further strengthened reflexivity.

### 2.6 Data analysis

The interviews were transcribed verbatim in Chinese by the first author (YL) and nine research assistants. YL checked all transcripts and corrected mistakes to ensure accuracy. The transcripts were then imported into NVivo (Version 12) for analysis.

The transcripts were analyzed following a process of reflexive thematic analysis (Braun and Clarke, 2021) underpinned by a critical realist ontology (Maxwell, 2012). Epistemological considerations for this analysis were contextualist. While the data provided grounding for the results, the interpretation and understanding of these data were influenced by the researchers’ perspectives and the social and cultural contexts in which the research was situated (Braun and Clarke, 2021). Both inductive and deductive orientations were adopted to analyze the data. The analytic meaning exploration was initially grounded in the data, but an ecological perspective (Doherty et al., 1998; Shek, 2006) and established findings deepened the analytic interpretation. Both semantic and latent coding were adopted to capture explicitly expressed and also hidden meanings of the data (Braun and Clarke, 2021).

The analysis process, although described sequentially for clarity, had an iterative nature. First, two researchers (YL and YZ) read and re-read the transcripts with audio recordings to become familiar with the data and then independently coded 9 out of 34 transcripts. Subsequently, YL and YZ discussed and compared codes, thoughts, and interpretations. The discussion outcomes were then shared with the entire research team, and the initial codes were reviewed and revised where necessary. Further to this, YL coded the remaining transcripts, collated the relevant codes into potential themes and subthemes, and reviewed, defined, and named the identified themes under the support of the research team through regular discussions and comparisons of the potential codes and themes. Finally, vivid and compelling extracts were selected and translated into English, and the report was produced.

## 3 Findings

**Figure 1:**
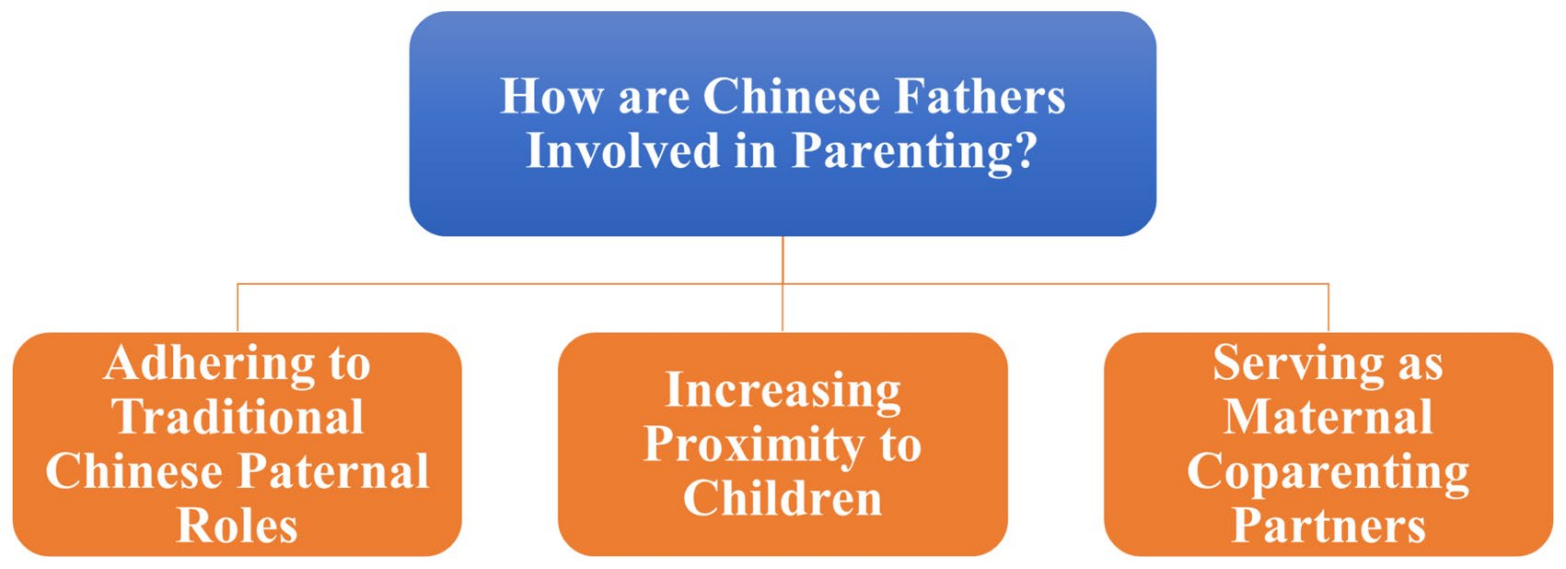
Thematic map.

**Figure 2:**
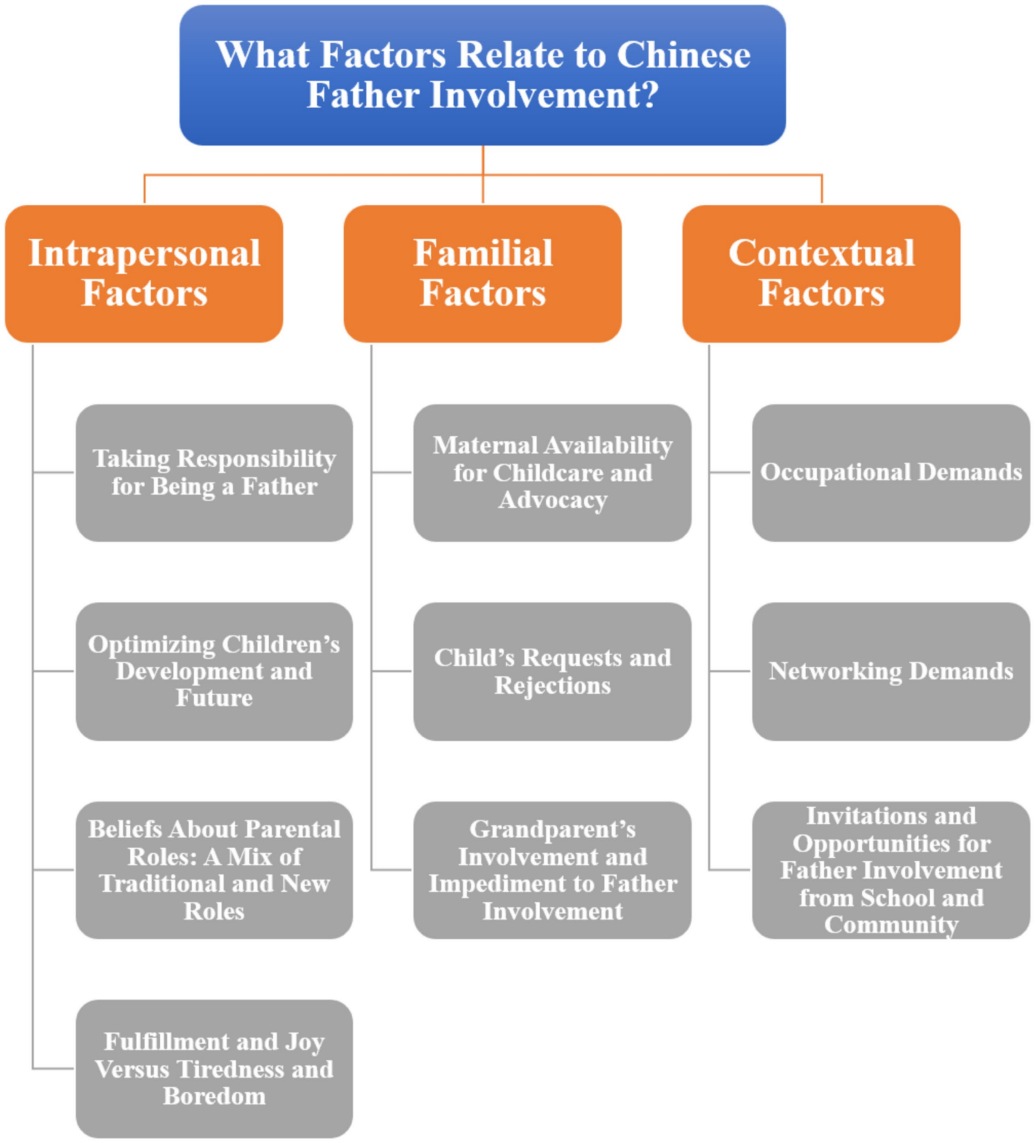
Thematic map.

The thematic analysis of the transcripts identified 35 codes, which were then collated into six key themes. The three main themes focused on how Chinese fathers are involved in parenting: adhering to traditional Chinese paternal roles, increasing proximity to children, and serving as maternal coparenting partners (see Figure 1). Chinese father involvement was related to intrapersonal, familial, and contextual factors (see Figure 2).

### 3.1 How are Chinese fathers involved in parenting?

#### 3.1.1 Theme 1: Adhering to traditional Chinese paternal roles

Chinese fathers described how they fulfill traditional paternal roles, including being educators, disciplinarians, and providers in children’s lives.

The educator role, which is a central part of a father’s role in traditional Chinese culture, was emphasized in almost every interview. Some fathers talked about their duties in imparting traditional social etiquette and moral codes, such as “being polite” and “respecting the aged and cherishing the young,” and cultivating children’s characters. In addition, fathers highlighted efforts to facilitate their child’s positive development across a number of domains, including academics, social skills, independence and autonomy, healthy habits, and hobbies and interests. Although their children were still in preschool, several fathers had already started teaching their children how to read, count, memorize ancient poems, and speak English. Some interviewees also signed up their children for extra-curricular activities and chose appropriate books, animations, and TV programs for their children. As Li said, “Like with some cartoons, sometimes I’ll check them out first. If I think they are okay, then I’ll let him watch them.” A few fathers suggested they considered, gathered information, and made decisions about their children’s learning and future development. Some of them described how they facilitated and encouraged children’s independence (e.g., feeding and dressing independently, sleeping alone) and development of social skills (e.g., encouraging peer interactions, dealing with bullying). Fathers also reported monitoring their own behaviors and emotions to set a good example for their children. “For example, in front of him (my child), [I] pay more attention to my words. Not using cuss words. Pronunciation is more standard when I talk,” Yang reported.

Most fathers indicated their role as disciplinarians who set rules and discipline children. As Wei said, “Comparatively speaking, if her mother or my parents (grandparents) cannot deal with the issue (the child’s misbehaviors), [they] would finally ask me to handle it (laugh).” In the process of disciplining children, some fathers highlighted positive parenting practices, including clear rules setting, calm instructions, communication, reward systems, and appropriate punishments. Yet, fathers also admitted to threatening, scolding, or even spanking their children when they misbehaved.

Financially supporting their children’s lives and meeting their material needs and requests were mentioned less by fathers. As well as providing food, clothes, and necessities, some fathers commented that they bought toys and good food to meet their children’s “reasonable” requests, even when it was against the mother’s wishes.

#### 3.1.2 Theme 2: Increasing proximity to children

In contrast to traditional Chinese fathers, who typically maintained more formal and distant relationships with their children, the interviewed fathers expressed how they provided companionship, supported their children, and engaged in caregiving activities.

All fathers described how they spent time with their children. Most of the activities were child-focused and playful (e.g., pretend play, watching TV, playing toys and video games, outdoor activities, sports, and exercise), whereas others had an enrichment or education focus (e.g., reading, playing chess, visiting museums and libraries). Talking to children about their lives was another activity many fathers reported. Zou suggested that he talk with his daughter about “her life in preschool” and “making friends.” In addition, Zhang described how he spent time with his daughter:

*When I’m at work, I make phone calls or video calls. Then, after work, [I] stay by her side. On days off, [I] either take the family out for fun or spend time with them at home. Basically, except for some social activities with friends, I spend the rest of my time with my wife and kid.*

Many fathers said they were involved in daily childcare to some extent, such as feeding, dressing, bathing, picking up from school, waking up, and putting to bed. Yet, the degree of involvement varied greatly. Some fathers were secondary caregivers who helped with the child’s care when they were at home [e.g., “Occasionally, when I have time, I play with her. Then, for eating, sometimes I help feed her.” (You)]. Others actively shared childcare with the children’s mother or grandparents, while a few fathers were primary caregivers responsible for the majority of childcare.

A few fathers indicated their roles in supporting and comforting children. For example, Qian suggested he told his child, “It’s not your fault.” and cheered him up when his child felt sad and wronged.

#### 3.1.3 Theme 3: Serving as maternal coparenting partners

Fathers described themselves as an important coparenting partner of the mother. Fathers explained how they cooperated with the child’s mother and shared the duties of childcare and housework at home: “Each person just shares one part, she does hers, I do mine, and then we [do it] together, right? Get this thing done well together.” (Shen). A variety of comments referred to discussions and decision-making between parents about parenting practices and the child’s education. As Jiang said, “Try to reach consensus. We must work out a direction to guide the child.” For some fathers, co-parenting also included providing the child’s mother with suggestions on how to raise the child in a better way. For example, Qin stopped the mother from scolding or spanking the child, and Xu reminded his partner to spend more time with the child than on her phone.

### 3.2 What factors relate to Chinese father involvement?

#### 3.2.1 Theme 4: Intrapersonal factors

##### 3.2.1.1 Taking responsibility for being a father

The word “responsibility” was often mentioned by fathers. They suggested that taking responsibility was important for a father. As Wang stated, “Since [I] am the child’s father, this is what [I] should do, absolutely.” Chen indicated that the responsibility motivates him to keep involved with this child: “I think it is responsibility. [I] even sometimes force myself. I should spend time with him. Not accompanying him is not okay.”

##### 3.2.1.2 Optimizing Children’s development and future

Fathers expressed their willingness to create a good environment, both physically and spiritually, to promote their children’s development. For example, Wu said, “I think we cannot leave everything to the child, but we should guide the child down a road and let him grow up healthily and happily, I think so.” Fathers also used the saying “Everything is for children” to explain why they were involved with their children, which suggested the high priority of children in fathers’ minds.

##### 3.2.1.3 Beliefs about parental roles: a mix of traditional and new roles

Participants held traditional beliefs about parental roles but also expressed modern and egalitarian beliefs.

###### 3.2.1.3.1 Inheritance of traditional Chinese parental roles

Almost half of the interviewees described a division of family and parental responsibilities consistent with traditional gender roles. Some fathers cited the popular Chinese proverb “Men are chiefly responsible for activity in society, while women are responsible for the home” to justify this division of labor. In these fathers’ view, a man should focus on his career and provide for the family, while a woman should be responsible for childcare.

In line with the gendered division of family responsibilities, a few fathers acknowledged that they were less patient and careful than mothers. Chen attributed these differences to the innate abilities of fathers and mothers, assuming mothers were better suited for childcare, such as feeding and dressing children. This view was echoed by Wei, who stated, “For example, her school organized some family trips. I usually let her mother involved in [these trips], because I feel the mother is more attentive to the child and take care of her more thoroughly.”

Beliefs in traditional father roles were also reflected in fathers’ recognition of their roles in *yang* and *jiao*. Some fathers emphasized the provider role in their beliefs and regarded it as the fundamental responsibility of being a father. “First, (let the child) stay warm and well-fed,” Zhou reported. Some fathers hoped to provide their children with better educational resources and material comfort and wanted to “leave more things (legacy) for the child in the future” (Sun) as a guarantee.

Almost all fathers indicated the importance of *jiao* or mentioned it when they were asked to define “good father.” In their beliefs, the scope of education was not limited to academics. Fathers cited one influential Chinese saying, “Teach by personal example as well as verbal instruction” (*yan chuan shen jiao*) to state what they should do in educating their children. In these fathers’ views, they should instruct children in traditional social etiquette and moral codes, such as “respect for seniority” (*zhang you you xu*) and “a sense of propriety, justice, honesty, and honor” (*li yi lian chi*), and also pass on their views of life and world. As Hua said, “Tell her what is right, what is wrong, what is good, what is bad, is not it?” Fathers also regard themselves as children’s role models, so they should be self-restrained and set an example for their children. For example, Sun commented: “I really want to establish images of being a good father, a good son, and a good husband for my son, but I find it…quite difficult.” Many fathers also believed that they played a critical role as mentors in shaping children’s character and good personalities (e.g., responsibility, courage, and determination), developing their social skills, and supporting them to understand society and the world. Fathers of sons also described the responsibility of showing how to be a man and sharing the personal experience of being a boy.

###### 3.2.1.3.2 Acceptance of new father roles

A few fathers expressed the view that fathers and mothers play similar roles in parenting. For example, Zhu commented:

*Because now, in fact, it is actually difficult to tell the gender difference, because now things that men and women can do, and duties and responsibilities [they] undertake can be the same, or even opposite or contrary, all possible. So now, there is no [gender difference].*

Fathers also acknowledged their awareness of mothers’ hard work and fatigue due to childcare and the need for help (e.g., “It is very tiring. Caring for the child is exhausting, much more tiring than work.” (Kong)). This may lead fathers to share childcare and housework at home: “She (the child’s mother) is usually quite busy when she goes back [home]. Of course, there are a lot of things [she] needs me to help her out.” (Shen).

In addition to supporting their partners, fathers also expressed their willingness to be actively involved and “accompany children growing up.” In their beliefs, most fathers considered spending time with their children as a crucial part of being a good father: “I think no matter how busy, [I] have to spend time with the child and play with the child.” (Han). In their view, fathers’ presence and companionship could benefit children’s mental health and personality, raise their emotional intelligence, and make them feel safe and secure. As Zhang highlighted, “Indeed, fathers hold a very important place in a child’s heart. Also, a father’s consistent presence can build good mental health in children.” Good communication with the child was also valued by fathers. For example, Jiang said, “Then, [a good father] can communicate with him, like friends. It’s good to be able to walk into his life and heart, rather than being superior.” This comment suggested the father’s willingness to establish an equal and friend-like relationship with his child. Other fathers also used “being a father and friend” or “being a teacher and friend” to describe the relationship with their children they pursued to develop.

The importance of caring about children was also emphasized in the interview. Fathers suggested that they should have a deep concern for their children’s physical and psychological well-being, development, academic learning, behaviors, and habits. One way to express their concerns for children was by meeting their requests: “How to attend to her? I feel it is to satisfy her under permitted conditions. This is what I should do. But for certain excessive demands, I feel [I] should not [satisfy her].” (Kong). A few fathers highlighted the importance of supporting, respecting, and helping children and comforting them when they are sad or ill. These included “spiritual support,” “hugging,” and “verbal encouragement.”

##### 3.2.1.4 Fulfillment and joy versus tiredness and boredom

Fathers described differing emotions they experienced as a result of being a father. Some highlighted feelings of joy and happiness in spending time with children and a sense of personal fulfillment from children’s progress, which motivated their involvement. As Zhou said:

*I feel that accompanying my child growing up, how to say, is a very meaningful or fun thing… Anyway, the time for companionships [with my child] is just a few years, so I do not think there are any other factors [making me involved], and I am actively involved.*

Fathers also acknowledged negative outcomes (e.g., tiredness, boredom) associated with parenting, which reduced their desire to spend time with the child: “Of course, sometimes I am a little lazy. That’s for sure. Because I mean, spending time with my child is very tiring.” (Kong). Positive and negative feelings could coexist and have a combined effect on levels of father involvement. For example, Qin reported, “You know, [I] very much want to take him (the child) out to play, but then [I] want to go back [home] early after playing, do you know?”

#### 3.2.2 Theme 5: Familial factors

##### 3.2.2.1 Maternal availability for childcare and advocacy

Levels of mother involvement were mentioned by fathers as affecting how they were involved with their children. Some fathers took a secondary caregiver role as their partners did the majority of childcare and spent more time with their children. In contrast, a few fathers had a primary caregiver role as their partners were busy with work and did not have much time for childcare. Li explained, “She comes back late and goes to work early because of her work. Then, basically, I am the one who does these things.”

Most fathers mentioned their partner requested or reminded them to spend more time with their children or complained about their low involvement. Chu said, “As long as I tell her that I will not go back home for dinner tonight, she will tell me, ‘You come back early.’” Other fathers also depicted similar situations when they worked overtime or had social activities. Also, mothers asked their partner to take care of their children (e.g., feeding, bathing), and reminded them of being more involved in parenting:

*Because I sometimes, to be honest, I sometimes play the computer, games and so on at home. Then, at this time, my wife would come and tell me, “Ah, cannot you spend the time with the daughter first after work?” I said, “Yes.” I put down things on my hands and spent time with my daughter first.* (Zhang).

Mothers also helped build father-child relationships. Wei described how the mother created an opportunity to involve him in play with their daughter:

*Sometimes, her mother would tickle her, and then she would tickle back, which is a reciprocal process of interactive play. Then, firstly, I would join in by tickling either her mother or her, and then wait for them to tickle me together. This is an approach for me to blend in.*

##### 3.2.2.2 Child’s requests and rejections

Child behavior also played a role in facilitating or hindering father involvement. Fathers reported that their children often came and asked for their companionship (e.g., to play, watch TV, read, and tell stories together). Wang described his daughter’s requests for support: “When she, for example, has conflicts and disputes with other kids, (she) then asks for your help. When she does not feel well, (she) seeks your comfort, right? Then when (she) feels down, (she) also talks to you.” Children-initiated interactions also brought happiness to fathers:

*Because you need to know, wow, two daughters, when (I) get up in the morning, wow, how good that feeling is when two daughters run into me. The feeling of the two running into me when I come home from work is so good, wow that I cannot even…* (Chu).

In contrast, a few fathers suggested that their child was closer to the mother or even actively refused their father’s involvement. Wu stated, “Basically, now my son always turns to his mom for eating and sleeping.” Chen said, “This (feeding and sleeping) is basically done by his mother. I do not have much patience in this aspect. Especially when sleeping, he does not turn to me.” Wei described his daughter’s rejection and fear of him: “In their (mother and daughter) play, as soon as I talk, the child quiets down and looks not that happy.”

##### 3.2.2.3 Grandparent’s involvement and impediment to father involvement

Some fathers mentioned that grandparents served as additional or even primary caregivers of their children: “The grandma provides more care in aspects of life.” (Feng) and “He is brought up by his grandma.” (Sun). Fathers explained that both parents were busy with work, and they needed support from grandparents. Shi viewed grandparents’ involvement as “at least a compensation” for their absence, and argued, “Then, we are not available sometimes, and hope the grandparents are able to substitute for us to take him out, like this, such as a school trip in autumn, such as some parent-teacher meetings in the school, just like this.”

Two fathers suggested that their own parents impeded their involvement in childcare as they distrusted their competence in parenting. Wei reported:

*Because as parents, which are our own parents, for ourselves, they always think that you cannot do this well and you cannot do that well. Then, it’s more likely to make the child sick, and more likely to make the child this, that, and the other. Then, their greater willingness is to help us take care of the child, and then we can have more time to handle our own affairs.*

#### 3.2.3 Theme 6: Contextual factors

##### 3.2.3.1 Occupational demands

Most fathers indicated that work, including long working hours, business trips, demands for full attention, and tiredness from work, prevented them from spending time with their children. Chu stated, “There are a lot of things [to do]. Sometimes it’s normal to come back home at eight, nine, or ten in the evening.” Shi commented, “After working all day, I’m also tired. To be honest, really tired. Right, sometimes after coming back from work, [I] have to do a little bit more to prepare for tomorrow’s work.” Fathers’ work requirements even made them put a higher priority on work than on childcare:

*For example, you make a date to take the child to the park today, and it just clashes with some of your own work arrangements. This situation can also happen, right? Then, we have to reschedule, and it definitely has to be subject to work arrangements.* (Chen).

##### 3.2.3.2 Networking demands

Social activities with friends and work colleagues were another factor affecting the time available to spend with their children:

*There are some, in fact, some obstacles. There are. For example, like myself, to be honest, being a worker, relatively, you have your own circle of friends and have some of your own social activities inside. To be honest, there are sometimes friends asking out drinking or friends asking out eating. Definitely, there are these kinds of things.* (Zhang).

##### 3.2.3.3 Invitations and opportunities for father involvement from school and community

Parent–child activities organized by their children’s schools (e.g., open days, trips) or by companies and community organizations facilitated father involvement with their children. As He said, “The school has some public activities, such as parent–child games, and I would participate. I sometimes put off work and come.”

## 4 Discussion

The aims of this study were to explore Chinese father involvement and to identify the factors that may play a role in facilitating or hindering their involvement from an ecological perspective. The results revealed that Chinese fathers both adhered to traditional paternal roles and increased proximity to their children while serving as maternal coparenting partners, and the intrapersonal, familial, as well as contextual factors were related to their involvement.

Consistent with Xu (2016) findings on Shanghai fathers who played multiple roles in parenting, these urban Chinese fathers not only fulfilled traditional paternal roles, namely *yang* (providing) and *jiao* (educating and disciplining) but also served as companions, caregivers, and supporters to increase proximity to their children and undertook co-parent roles. While these Chinese fathers assumed both traditional and contemporary paternal roles, a comparison with Western and other Chinese studies revealed subtle differences in parenting roles fathers undertook or prioritized.

When we compare the three traditional Chinese paternal roles, it is informative that the educator and disciplinarian roles were mentioned by the majority of fathers, while the provider role was less identified. Unsurprisingly, most fathers described their roles as educators and disciplinarians in their children’s lives, since *jiao* (educating and disciplining) has consistently been seen as Chinese fathers’ critical parenting roles. Fathers’ active engagement in teaching and disciplining their children has frequently been highlighted in previous Chinese research (Li, 2020b; Liong, 2017; Xu, 2016), whereas this aspect has seldom been a focal point in Western contexts (Li et al., 2021). However, the reason the provider role was mentioned less in the interview deserves further discussion and investigation. One possible explanation is that financial provision was not seen as part of “involvement” that may require direct interaction in the fathers’ view. Another possibility is that fathers have seen the provider as an “assumed role,” so they did not stress it in particular (Chuang and Su, 2008).

The interviewed fathers also undertook contemporary paternal roles, including companions, caregivers, and supporters, to increase proximity to their children. All fathers described how they spent time with their children. This is in line with previous interviews with Hong Kong fathers, who described their roles in playing and communicating with their children (Lau, 2016). Surprisingly, many fathers indicated that they were involved in caregiving their children, although the degree ranged from assistants to primary caregivers. This finding aligns with the observed increase in the amount of daily time Chinese fathers of children aged 0–6 years spent on family caregiving, which rose from 0.68 h in 2008 to 0.92 h in 2017 (Du et al., 2018). Today’s Chinese fathers somewhat share everyday childcare activities (Li, 2018), which was traditionally regarded as mothers’ nurturing responsibility (Cao and Lin, 2019). However, only a few fathers expressed how they were involved in supporting and comforting their children. No one reported how they showed affection and made children feel loved, which is inconsistent with both Western (Summers et al., 2006) and Chinese studies (Lau, 2016; Li and Jankowiak, 2016). Traditionally, Chinese fathers are expected to display love and affection in subtle and implicit ways so as to maintain their authority in strictly disciplining their children (Li and Lamb, 2015). Some Chinese fathers today, like their Western counterparts, are more comfortable expressing affection (Lau, 2016; Wang and Keizer, 2024), while others would still be conservative in displaying love to their children through physical affection (Li, 2020b; Liu and Zheng, 2021). The fathers interviewed were from Fuzhou City. In contrast to other regions of China, such as Shanghai, Chengdu, and Chongqing (Wang and Keizer, 2024), men in Fuzhou are more adherent to traditional gender roles, potentially making them less likely to express affection to their children openly. Such disparities indicated that the parenting roles that Chinese fathers play may vary substantially across different regions.

Besides, many fathers described how they served as maternal coparenting partners, including sharing in childcare, housework, and decision-making, and positively influenced their partners’ parenting practices. This description indicated that some Chinese fathers, like their counterparts in Western (Gervais et al., 2020), played an important role in supporting their partner and coparenting their children. It is challenging to raise children alone. As a teammate of the mother, fathers can also facilitate decision-making and help prevent errors in parenting practices.

Importantly, fathers’ responses in this study provided some explanation for the variation in their level of involvement in parenting. Chinese fathers identified intrapersonal, familial, and contextual factors related to their involvement with their children, which supported that multi-layered factors jointly influence how fathers are involved with their children from an ecological perspective (Doherty et al., 1998; Shek, 2006).

Intrapersonal factors included fathers’ personal beliefs about fatherhood and perceptions of parenting. Fathers highlighted the importance of taking responsibility for being a father and optimizing their child’s development and future. These beliefs appeared to motivate them to engage in childrearing. Similar views were also found in other contexts. For instance, fathers in urban Tajikistan indicated that “responsibility” was a source of motivation for them to raise their children well (Kasymova and Billings, 2018), and Quebec fathers suggested their children’s well-being was a high priority in their decision-making and lives (Gervais et al., 2020). The beliefs about paternal roles held by Chinese fathers in this study encompassed both traditional and more modern and egalitarian beliefs. For example, some fathers still referenced the popular Chinese proverb “Men are chiefly responsible for activity in society, while women are responsible for the home” to rationalize the gendered division of labor, in line with the patriarchal traditions endorsed by Confucian ideology. In contrast, others believed that fathers and mothers play similar roles in parenting and expressed the willingness to support their partner and participate in childrearing. Chinese fathers’ mixed beliefs are consistent with both traditional and contemporary paternal roles they played when involved with children, which suggests the close relationships between fathers’ beliefs and behaviors. Yet, it should be noted that no father mentioned the caregiving role when they defined “good father,” while many fathers were actually involved in daily childcare. This gap between fathers’ beliefs and behaviors might suggest that Chinese fathers may not regard childcare as their responsibility, but contextual factors, such as requests from their partner, might involve them in caregiving. In addition to fathers’ beliefs, their perceptions of parenting (e.g., fulfillment, tiredness) affected their desire to be involved with their children.

Familial factors affecting father involvement comprised the roles of mothers, children, and grandparents. Consistent with the conceptual model of influences on fathering derived by Doherty et al. (1998), mothers’ availability for childcare and their requests, complaints, and support were related to levels of father involvement. Fathers only reported how their partner facilitated their involvement in parenting and childcare but did not mention any discouragement or limitation from mothers, also known as gate-closing behaviors, found in the previous research (Wang et al., 2019; Zou et al., 2019). This disparity may be due to social desirability in that fathers would not like to discuss conflicts with their partner. Research methods allowing anonymity might be needed to explore the possibility of maternal gate-closing behaviors in the Chinese context.

Apart from mothers, other family members’ behaviors also played a role in influencing father involvement. Chinese fathers in this study reported the impacts of children’s requests for and rejections of their involvement in fathering. This finding enhanced our understanding of the influence of children on the father, which has hitherto received scant attention in fatherhood research (McLaughlin and Muldoon, 2014). In particular, fathers also described the influence of grandparents on their fathering, which is less discussed in Western research, such as Doherty et al. (1998) conceptual model. Despite an increase in nuclear families in contemporary China (Li and Lamb, 2013), it is common for grandparents to participate in childcare following the birth of a child, which is attributed to a scarcity of affordable public childcare options and a high rate of female labor participation (Li et al., 2021). 73.5% of families in this interview had grandparents living in the same household. Although the fathers acknowledged the need for grandparent support, some were also wary that grandparents may impede or hinder them from being involved with their children. Thus, grandparents may be another “gatekeeper” in childrearing. When discussing familiar factors related to father involvement, the role of grandparents should be included in further studies.

Finally, broader contextual factors identified by fathers included occupational demands, networking demands, and invitations as well as opportunities for parental involvement from school and community. Fathers experienced long working hours, business trips, and tiredness from work, which prevented them from participating in childcare and parenting. The influence of work on father involvement was also found in previous research in China (Lau, 2016; Wu et al., 2014) and other countries (Daly, 1996; Kasymova and Billings, 2018). Additionally, social activities with friends and colleagues sometimes took up fathers’ time with their children. Parent–child activities organized by their children’s school or other organizations, however, played a role in engaging fathers, which confirmed the argument of Hoover-Dempsey and Sandler (1997) that general opportunities, invitations, and demands from children’s schools facilitate parental involvement.

Our research is one of the few studies that use qualitative methods to explore Chinese father involvement using a sample of fathers from Mainland China. It had a good sample size. The research provided a rich insight into Chinese fathers’ subjective experiences of being involved with their children and their views about the factors related to their involvement. The study, however, has several limitations. There were three sampling issues that limited the generalizability of the findings to the entire Chinese population. First, the findings were based on the responses of 34 educated, urban, self-selected fathers. The themes identified might, therefore, be more reflective of involved fathers who volunteered to participate in a fatherhood study than less educated fathers or those living in rural China. Second, the sample of this qualitative study was comprised only of married fathers. As such, the identified themes cannot be generalized to divorced or single fathers. Nonetheless, given the low divorce rate in China, we argue that this sample is valuable. Third, the fathers were recruited via a survey study that included both parents, suggesting that they likely maintain a positive relationship with the mother or, at the very least, share a consensus with her about the importance of parenting and child development. This sampling approach further limited the applicability of the findings. Further research should sample fathers from different groups and socioeconomic status (e.g., nonresident fathers, single fathers, low-income fathers, less-educated fathers, rural workers) to determine whether these findings can be applied to other groups of fathers or the Chinese father population in general. In addition, this study, by design, relied only on self-report data from fathers. This was intentional in order to capture the nuanced opinions of fathers. However, further research should include reports of mothers, children, or even grandparents to better understand Chinese father involvement and improve research credibility. Research that triangulates data from multiple sources may be particularly beneficial in uncovering the interplay between the multiple factors that influence Chinese father involvement. Furthermore, the interview guide did not include dedicated questions exploring the potential effects of grandparents on father involvement. This omission arose because the development of the interview guide relied on existing fathering literature, such as Doherty et al. (1998) conceptual model, which largely overlooks the role of grandparents. Future research could incorporate questions about grandparents’ roles in fathering to deepen the understanding of their potential influences.

## 5 Conclusion and implications of the research

The present study gave voice to Chinese fathers to explore their experiences of involvement with their children and what factors relate to their involvement. The findings indicate that fathers both fulfilled traditional paternal roles and increased proximity to their children while serving as maternal coparenting partners, and the intrapersonal, familial, as well as contextual factors were related to their involvement.

The intrapersonal, familial, and contextual factors identified in the present study supported that multi-layered factors jointly influence how fathers are involved with their children (Doherty et al., 1998; Shek, 2006). However, different from Doherty et al. (1998) conceptual model developed in Western contexts focusing solely on child and mother characteristics, the role of grandparents in father involvement was highlighted in Chinese fathers’ narrative. This discrepancy highlights how cultural norms and family structures shape family dynamics. Doherty et al. (1998) conceptual model, premised on nuclear family assumptions, may not apply to the contexts where multigenerational living is common, such as in China and many other Asian countries. In such multigenerational families, grandparents often play an influential role in family interactions and parenting practices. Future research should carefully consider the potential impact of grandparents in these cultural contexts to better understand how multigenerational dynamics influence father involvement and family relationships.

Importantly, the multiple factors related to father involvement also can provide important clues for researchers, practitioners, and politicians to tailor supports to facilitate father involvement. Family intervention and programs could focus on addressing fathers’ beliefs and responsibilities, as well as enhancing the support from mothers, grandparents, and children, to strengthen fathers’ commitment to parental roles. Additionally, the findings highlighted that long working hours, business trips, and work-related social activities might limit fathers’ time available to spend with their children. The implementation of family-friendly policies, such as flexible working hours, paid parental leave, or support for work-life balance, might be beneficial for increasing father involvement.

## Data Availability

The raw data supporting the conclusions of this article will be made available by the authors, without undue reservation.

## Appendix

**Table 1:** Interview guide.

Interview questions
1. How do you define “good father”?
2. In your opinion, how important (if at all) is the role of fathers on children?
*Follow up: Why do you believe that? Can you tell me more?*
3. How are the roles of fathers and mothers different or the same in parenting children?
4. How are you involved in your child’s life?
*Prompt: Can you give me some examples or more details?*
5. What kind of things would make you more likely to be involved in parenting your child?
6. What kind of things would make you less likely to be involved in parenting your child?
7. How would your wife/partner define “good father”?
*Follow up: Why do you believe that?*
*Prompt: What did your wife/partner say and do that demonstrate that?*
8. Do you agree/disagree with that view?
9. How does that influence your role as a father?
10. Is there anything your wife/partner says and does that influence your role as a father?
